# Varying Clinical Phenotypes of Mitochondrial DNA T12811C Mutation: A Case Series Report

**DOI:** 10.3389/fmed.2022.912103

**Published:** 2022-07-04

**Authors:** Qingdan Xu, Ping Sun, Chaoyi Feng, Qian Chen, Xinghuai Sun, Yuhong Chen, Guohong Tian

**Affiliations:** ^1^Department of Ophthalmology, Eye and ENT Hospital, Fudan University, Shanghai, China; ^2^NHC Key Laboratory of Myopia, Chinese Academy of Medical Sciences, and Shanghai Key Laboratory of Visual Impairment and Restoration, Fudan University, Shanghai, China; ^3^State Key Laboratory of Medical Neurobiology, Institute of Brain Science, Fudan University, Shanghai, China

**Keywords:** Leber hereditary optic neuropathy, mitochondrial disorder, mitochondrial DNA, ophthalmoplegia, optic atrophy

## Abstract

The T12811C mitochondrial DNA (mtDNA) mutation has been reported in Leber hereditary optic neuropathy (LHON) previously, with vision loss as the main manifestation. The involvement of other organ systems, including the central and peripheral nervous system, heart, and extraocular muscles, has not been well described. This case series report investigated four patients with T12811C mtDNA mutation, verified through a next generation sequencing. Two male patients presented with bilateral subacute visual decrease combined with involvement of multiple organ systems: leukoencephalopathy, hypertrophic cardiomyopathy, neurosensory deafness, spinal cord lesion and peripheral neuropathies. Two female patients presented with progressive ptosis and ophthalmoplegia, one of whom also manifested optic atrophy. This study found out that patients harboring T12811C mtDNA mutation manifested not only as vision loss, but also as a multi-system disorder affecting the nervous system, heart, and extraocular muscles.

## Introduction

Leber hereditary optic neuropathy (LHON) is a maternally inherited ocular disease leading to painless, progressive, bilateral loss of central vision in young adults, predominately affecting males (1–5). Although visual dysfunction is the most common symptom of LHON, other systemic abnormalities can also occur. These include dystonia, Parkinsonism, extra-pyramidal motor symptoms, peripheral neuropathy, cardiac conduction abnormalities, spinal cord disease, and skeletal muscle abnormalities (6–13). The maternal transmission indicates that mutations in the mitochondrial DNA (mtDNA) play key roles in the development of LHON. Three mtDNA variants are considered as primary mutations causing LHON, namely, ND4 G11778A, ND1 G3460A, and ND6 T14484C (14, 15). These three mtDNA mutations account for over 95% of LHON cases worldwide, while the remaining LHON cases may be caused by other variants (4).

The T12811C (Y159H) mutation is located in highly conserved residues in ND5 that encodes the subunit of respiratory chain complex I (16). This missense mutation results in the substitution from tyrosine to histidine. In previous investigations, Huoponen et al. reported that the ND5 T12811C mutation acted as a secondary mutation that increased the penetrance of the ND4 G11778A mutation in LHON (17). Here, we investigated the clinical phenotypes of the T12811C mutation in four Chinese patients diagnosed with LHON and other systemic diseases. The detailed clinical manifestations and genetic characteristics were evaluated. Our study reveals the genetic heterogeneity of mitochondrial diseases and expands the phenotypic spectrum of T12811C mutation.

## Materials and Methods

### Patients

This study was approved by the Institutional Review Board of Eye, Ear, Nose, and Throat Hospital of Fudan University (KJ2011-04). Written consent forms were obtained from all participants or their guardians. Four Han Chinese patients who had ND5 T12811C mutation were enrolled at the Neuro-Ophthalmology Division of the Eye, Ear, Nose, and Throat Hospital of Fudan University, Shanghai, China.

### Clinical Investigation

Detailed medical histories were recorded for all the patients, especially the family histories as well as histories of smoking and drinking. All the patients underwent complete neuro-ophthalmologic evaluations including assessment of best-corrected visual acuity (BCVA), color vision using Ishihara plates, relative afferent papillary defect (RAPD), slit-lamp biomicroscopy, fundoscopy, Goldmann/Octopus visual field perimetry, and spectral-domain optical coherence tomography (OCT). Other ancillary tests focused on neurological system, heart, and hearing were performed according to the patients’ different symptoms. Ophthalmic examinations and neurological evaluations were also conducted for all the parents.

### Genetics Analysis

The total DNA was isolated from peripheral blood of the four patients. For case 1, case 3, and case 4, next generation sequencing was utilized and a panel of 194 optic-atrophy associated genes were sequenced using the Illumina HiSeq 2000 (Illumina, Inc., San Diego, CA, United States) sequencing system. The panel covered the whole mtDNA genome and the nucleus genes related to mitochondrial disorders. The average coverage depth was 300 times. Ninety nine percent of the target region received 20 times coverage or more. For case 2, a small panel covering nine optic-atrophy associated nucleus genes (*RTN4IP1*, *YME1L1*, *OPA1*, *OPA3*, *TMEM126A*, *DNM1L*, *ACO2*, *WFS1*, *CISD2*) and the whole mtDNA genome were sequenced.

## Results

### Case 1

**FIGURE 1:**
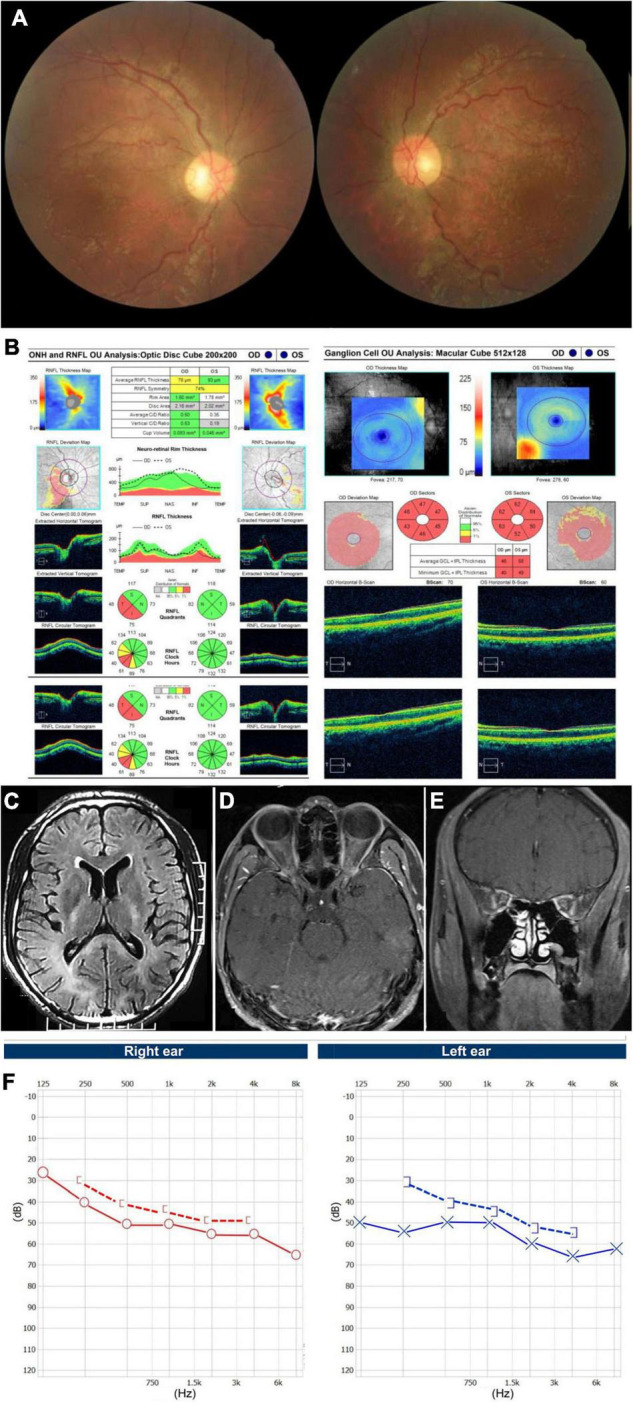
Accessory examinations of case 1. **(A)** Fundus photos showed diffuse pallor in the right eye and temporal pallor in the left eye, with tortuous retinal vein bilaterally. **(B)** OCT showed thinning of inferior-temporal RNFL in the right eye and severe GCIPL loss bilaterally. **(C)** Axial T2 Flair-weighted MRI of the brain showed hyperintense signal changes involving the posterior limbs of internal capsule, occipital lobes and corpus callosum indicating leukoencephalopathy. **(D)** Axial and **(E)** coronal T1-weighted MRI of orbits with gadolinium and fat suppression showed bilateral optic nerve slight enhancement. **(F)** Audiography showed bilateral neurosensory deafness.

A 25-year-old man presented with painless vision loss in both eyes for 1 month. He noticed he could not see clearly in the right eye for 10 days and when first examined, both eyes’ visual acuity was abnormal. He was diagnosed with hypertrophic cardiomyopathy 7 years ago. He complained of hearing loss for years and the pure tone audiometry demonstrated moderate neurosensory hearing loss for both ears. He had no family history of similar diseases and denied smoking or drinking alcohol. The BCVA was hand motion in his right eye and 20/400 in his left eye. Color vision was 0/8 in both eyes. There was no RAPD in the right eye. Funduscopic examination showed diffuse pallor in his right eye and temporal atrophy of optic disc in his left eye, with bilateral tortuous retinal vein (Figure 1A). Goldmann visual field tests revealed central scotomas in both eyes. OCT demonstrated thinning of inferior-temporal retinal nerve fiber layer (RNFL) in the right eye and severe ganglion cell-inner plexiform layer (GCIPL) loss in both eyes (Figure 1B). T2 Flair-weighted magnetic resonance imaging (MRI) of the brain showed hyperintense signal changes involving the posterior limbs of internal capsule, occipital lobes and corpus callosum indicating leukoencephalopathy (Figure 1C). T1-weighted MRI of the orbits with gadolinium and fat suppression showed slight enhancement of the bilateral optic nerves in both axial (Figure 1D) and coronal (Figure 1E) views. The audiography demonstrated bilateral neurosensory deafness (Figure 1F). Genetic testing results showed the T12811C mutation with the heteroplasmy at the level of 99.81%. Another variant of G3946A in ND1 with the heteroplasmy of 16.45% was also detected. The patient was diagnosed with Leber plus syndrome and prescribed coenzyme Q 10 together with idebenone. His visual acuity stabilized at Snellen 20/400 for both eyes after 1 year of follow-up (Table 1).

**TABLE 1:** Clinical manifestations of the four cases.

	Sex	Age (year)	Presenting symptoms	Neuro-ophthalmologic examination						Other systems involvement	MRI brain/orbit
				BCVA	Color	Fundus	Visual field	Eye movement	OCT		
Case 1	M	25	Visual decrease Heart disease Hearing loss	HM OD	0/8 OD	Diffuse pallor, tortuous retinal vein OD	Central scotoma OD	Normal OD	Thinning of inferior-temporal RNFL OD and severe GCIPL loss OU	Leukoencephalopathy Hypertrophic cardiomyopathy Neurosensory deafness	Leukoencephalopathy, bilateral optic nerve atrophy
				20/400 OS	0/8 OS	Temporal pallor, tortuous retinal vein OS	Central scotoma OS	Normal OS			
Case 2	M	58	Visual decrease Low limb paraplegia Numbness of arms and legs	20/200 OU	0/8 OU	Temporal pallor OU	Central scotomas OU	Normal OU	Thinning of RNFL and severe GCIPL loss OU	Peripheral neuropathies Spinal cord lesion	Bilateral optic nerve atrophy without spinal cord lesion
Case 3	F	32	Progressive ptosis Eye movement disorders Visual decrease	20/60 OD	4/8 OD	Temporal pallor OD	Normal OD	Ptosis, horizontal and vertical movement deficient OU	Thinning of RNFL OD and severe GCIPL loss OU		Thinning of extraocular muscles without other brain lesion
				20/30 OS	6/8 OS	Normal OS	Normal OS				
Case 4	F	16	Progressive ptosis Eye movement disorders	20/20 OU	8/8 OU	Normal OU	Normal OU	Ptosis, horizontal and vertical movement deficient OU	Normal OU		Thinning of extraocular muscles without other brain lesions

### Case 2

**FIGURE 2:**
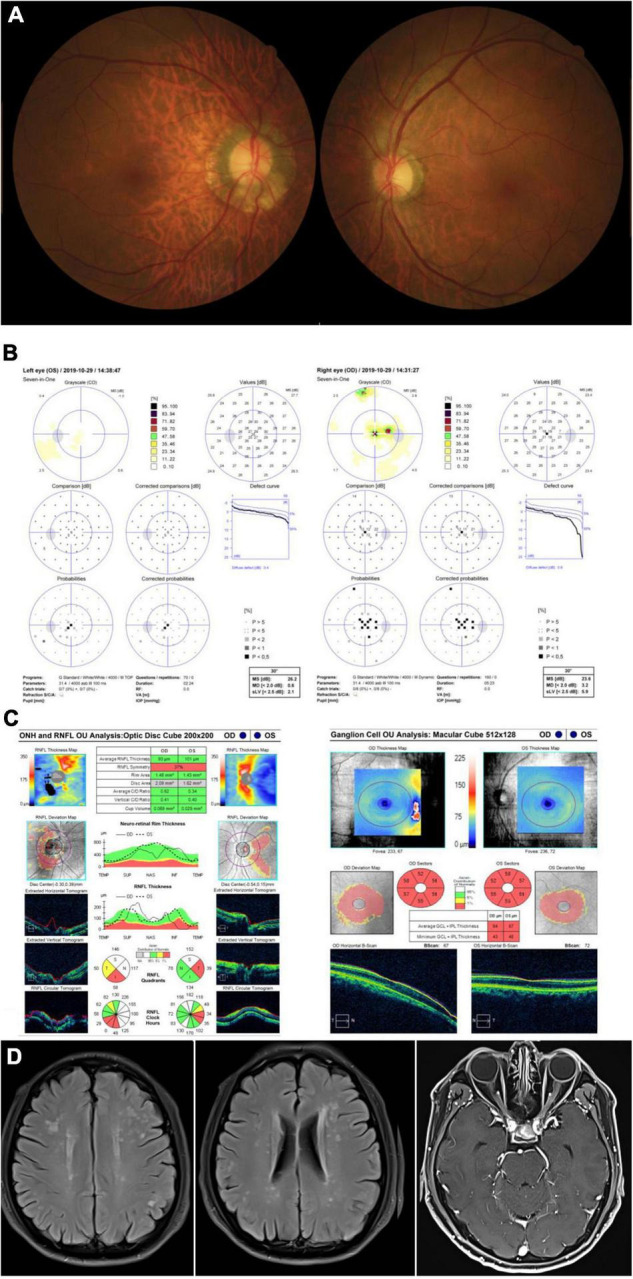
Accessory examinations of case 2. **(A)** Fundus photos showed temporal pallor of optic disc bilaterally. **(B)** Octopus visual fields showed central scotomas bilaterally. **(C)** OCT revealed inferior-temporal RNFL thinning in the right eye and temporal RNFL thinning in the left eye, with significant thinning of the GPICL bilaterally. **(D)** Brain MRI showed scattered T2 Flair-weighted hyperintense signal and orbital T1-weighted MRI imaging after contrast showed bilateral optic nerve atrophy without enhancement.

A 58-year-old man presented with bilateral progressive visual decrease within 2 months, accompanied by weakness and numbness of his legs. He noticed both eyes blurry in the morning without periorbital pain or headache. He did not have any family history of similar diseases. He denied taking any neurotoxic medication, smoking or drinking alcohol. The BCVA was 20/200 and color vision was 0/8 in both eyes. The fundus showed temporal pallor of the bilateral optic discs (Figure 2A). The neurological examination only showed bilateral peripheral neuropathies. Muscle strength was level IV but with paresthesia of both arms and legs. Octopus visual fields showed central scotomas in both eyes (Figure 2B). The OCT showed inferior-temporal RNFL thinning in the right eye and temporal RNFL thinning in the left eye, with significant thinning of the GCIPL in both eyes (Figure 2C). The brain MRI showed scattered T2 Flair-weighted hyperintense signal and orbital MRI only showed bilateral optic nerve atrophy without enhancement in T1-weighted imaging after contrast (Figure 2D). A small pituitary tumor was reported which was irrelevant to optic neuropathy. There was no reported spinal cord lesion. Genetic testing revealed the T12811C mutation with the heteroplasmy at the level of 98.93%. The patient was diagnosed with Leber plus syndrome and prescribed cobamamide together with coenzyme Q 10 and idebenone. His visual acuity was stabilized at Snellen 20/200 for both eyes during follow-up (Table 1).

### Case 3

**FIGURE 3:**
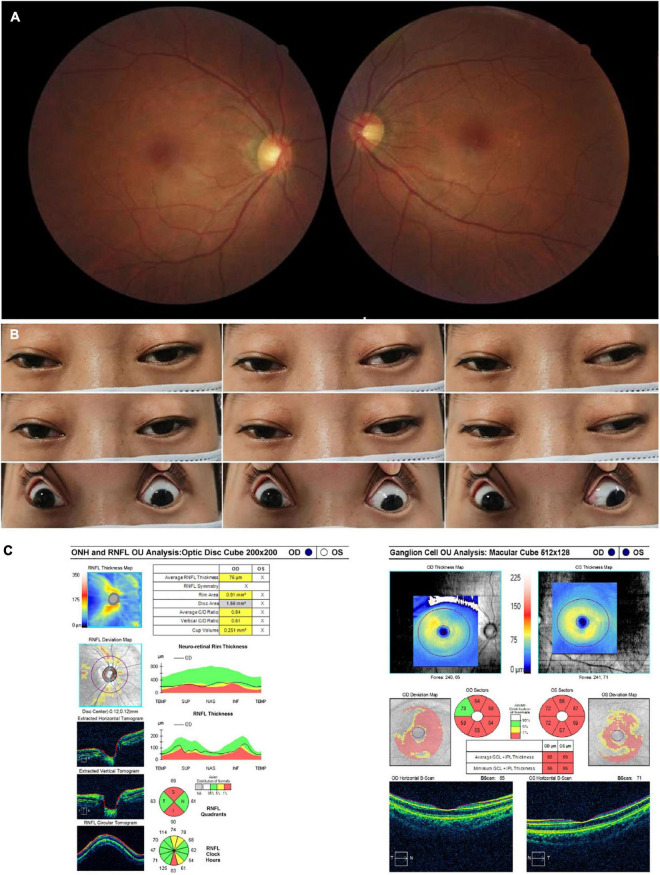
Accessory examinations of case 3. **(A)** Fundus photos showed temporal pallor in the right eye. **(B)** Nine cardinal eye positions showed impaired mobility in both eyes, including adduction, abduction, elevation, and depression. **(C)** OCT showed thinning of RNFL superiorly and inferiorly in the right eye, with diffuse thinning of GCIPL bilaterally. The RNFL thickness of the left eye was not available due to poor cooperation.

A 32-year-old woman complained of progressive bilateral ptosis and strabismus since she was 20 years old. She underwent strabismus surgery 10 years ago. She had no family history of similar diseases and denied smoking or drinking alcohol. Tests for ocular myasthenia gravis, including acetylcholine receptors antibody, single-fiber electromyography, repetitive nerve stimulation, and chest computed tomography scan were all negative. The BCVA was 20/60 in her right eye and 20/30 in her left eye. Color vision was 4/8 in her right eye and 6/8 in her left eye. Funduscopic examination revealed temporal pallor of the optic disc in her right eye (Figure 3A). Eye movement was impaired in both eyes, including adduction, abduction, elevation, and depression (Figure 3B). The OCT demonstrated thinning of the RNFL superiorly and inferiorly in the right eye and diffuse bilateral thinning of GCIPL (Figure 3C). The brain and orbital MRI showed thinning of extraocular muscles without other brain lesions. Genetic testing results detected the T12811C mutation only with the heteroplasmy at the level of 99.49%. No gene fragment deletion that was responsible for chronic progressive external ophthalmoplegia (CPEO) syndrome was found. She was finally diagnosed with mitochondrial disease with LHON and CPEO syndrome and prescribed coenzyme Q 10 together with idebenone (Table 1).

### Case 4

**FIGURE 4:**
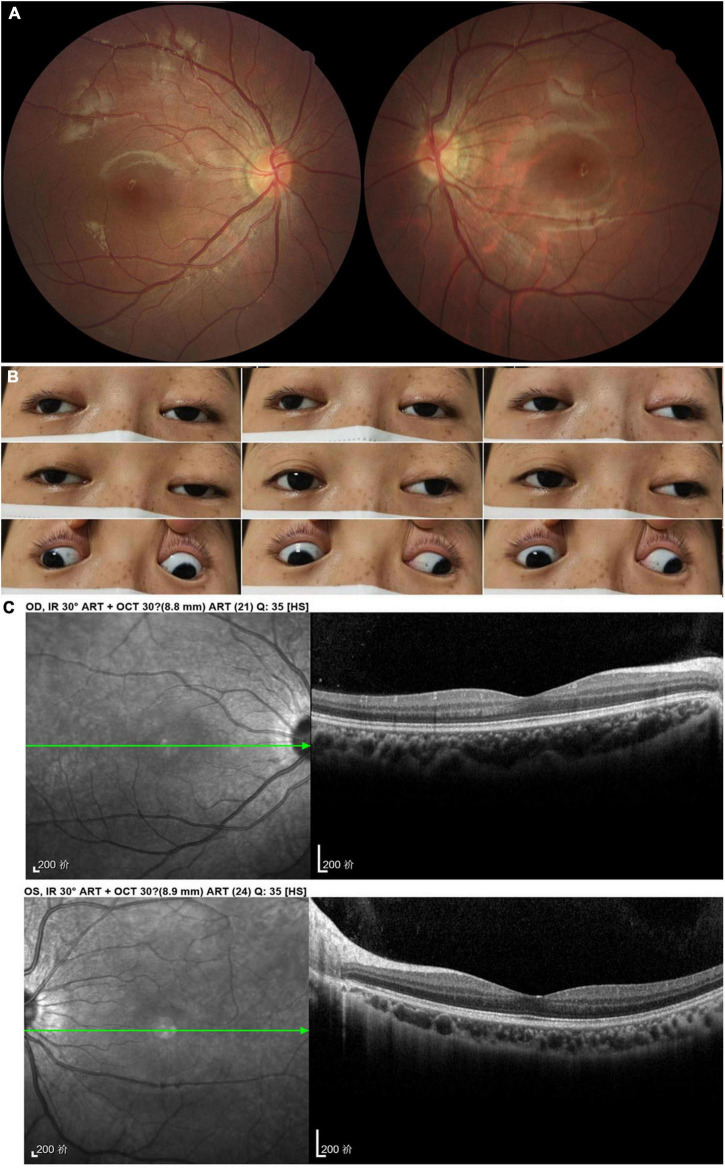
Accessory examinations of case 4. **(A)** Fundus photos showed normal optic disc and retina bilaterally. **(B)** Nine cardinal eye positions revealed adduction, elevation, and depression were moderately reduced bilaterally, and abduction was slightly reduced in the right eye and almost full in the left eye. **(C)** OCT showed normal retina thickness and macular structure bilaterally.

A 16-year-old woman presented with bilateral droopy eyelids and extraocular muscle weakness for about 5 years. She had no family history of similar diseases and denied smoking or drinking alcohol. The BCVA was 20/20 and color vision was 8/8 in both eyes. The fundoscopy revealed normal optic discs without retinopathy in both eyes (Figure 4A). Ophthalmic examination only revealed bilateral ptosis and exotropia. Adduction, elevation, and depression were moderately reduced in both eyes. Abduction was slightly reduced in the right eye and almost full in the left eye (Figure 4B). The OCT revealed normal retina thickness in both eyes (Figure 4C). The brain and orbital MRI showed thinning of extraocular muscles without other brain lesions. Genetic testing results showed the T12811C mutation with the heteroplasmy at the level of 99.94%. No gene fragment deletion which was usually found in CPEO patients was detected in this patient. She was finally diagnosed as ophthalmoplegia with mitochondrial disease and prescribed coenzyme Q 10 together with idebenone (Table 1).

## Discussion

This case series study reported the multi-system phenotypes caused by the T12811C mutation. Our patients with an identical T12811C mutation in ND5 showed different clinical symptoms: vision loss, hypertrophic cardiomyopathy, hearing loss, paraplegia, ptosis, and extraocular muscle weakness. These clinical findings showed the broad phenotypic spectrum of the single point mutation of T12811C in mtDNA. It involved multiple systems, such as optic atrophy, cardiac abnormality, leukoencephalopathy, cochlea lesion, spinal cord lesion, peripheral neuropathies, and extraocular ophthalmoplegia.

The pathophysiology of LHON is complex. It typically manifests as an acute or subacute loss of central vision, a result of optic atrophy secondary to severe damage of retinal ganglion cells (18). The accompanying abnormalities, mainly neurological disorders, have also been reported in some patients (19). Three particular variants of G11778A, T14484C, G3460A in mtDNA are confirmed as primary mutations and explain over 95% of LHON patients worldwide (20–22). T12811C was initially published as a secondary LHON-associated mutation exacerbating the visual loss of LHON (16, 17). Recently, T12811C was reported as a primary mutation for the first time in one case, who presented as isolated LHON (23). Our group of patients manifested as multi-system disorders, thereby expanding the phenotypes in association with the T12811C mutation.

**FIGURE 5:**
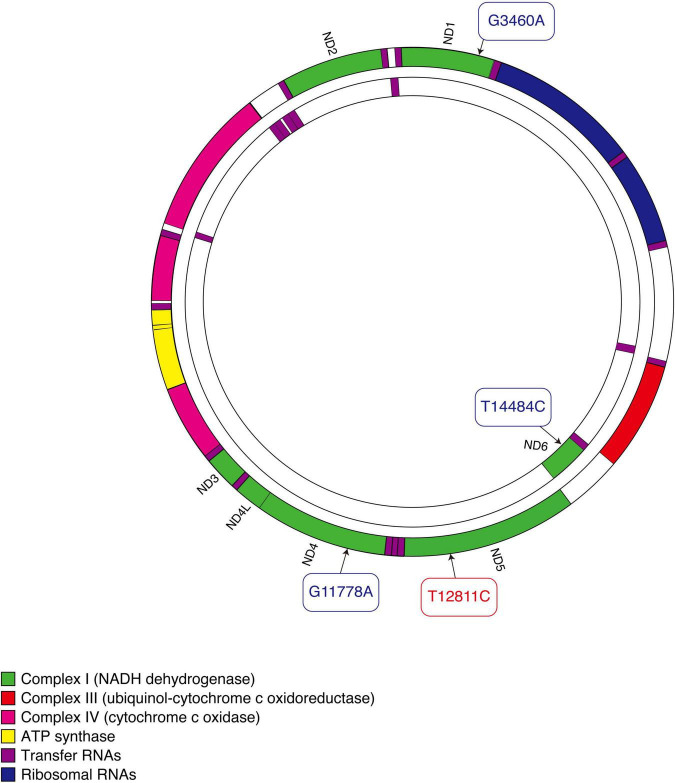
Schematic diagram of mitochondrial DNA. Three primary mutations causing LHON are shown in blue. The mutation that our patients carried is shown in red.

The T12811C mutation changes an evolutionarily conserved tyrosine to a histidine in the transmembrane region of ND5, the core subunit of complex I (16) (Figure 5). This mutation was predicted to alter the structure of the transmembrane region of the ND5 protein (24). ND5 plays an important role in the stability and activity of complex I (25). Researches have revealed that ND5 protein synthesis is the rate-limiting step for complex I activity and the highest rate of ND5 protein synthesis is just sufficient to maintain a normal respiratory rate (26–28). This may explain the experimental results that defects in ND5 can lead to a significant decrease in rates of mitochondrial respiration (28, 29). Hence, we consider the mutation T12811C in the ND5 gene to be pathogenic and responsible for the clinical phenotypes in our patients.

Furthermore, previous studies suggested that mutations in the ND5 gene were more likely to be associated with an extended phenotype rather than isolated visual dysfunction (30). Diseases caused by the mutations in ND5 would exhibit various degrees of clinical heterogeneity, such as Leigh syndrome (31), Idiopathic Parkinson’s disease (32), mitochondrial encephalomyopathy, lactic acidosis and stroke-like episodes (MELAS) (33), myoclonic epilepsy with ragged-red fibers (MERRF) (34), and hypertrophic cardiomyopathy (35). Strikingly, neurological and muscular disorders are the main problems caused by the mutations in ND5 gene, which were also observed in our patients. Besides, deafness (36), paraplegia (37), ptosis (38), strabismus (39), and ophthalmoplegia (40) have also been occasionally seen in patients with mutations in the ND5 gene. To the best of our knowledge, our study is the first report describing ophthalmoplegia as the only symptom in such patients. Since no gene fragment deletion responsible for CPEO syndrome was found in our cases, ophthalmoplegia is most likely a phenotype of T12811C. Unfortunately, the biopsy of muscle was rejected in the two patients with opthalmoplegia according to patients’ will. The histochemistry of COX and SDH together with detected mtDNA variants in muscle sample would be a great help in the diagnosis of mitochondrial CPEO.

Some researchers have proposed hypotheses explaining how the same gene mutation can cause different phenotypes. Patients with mitochondrial diseases harbor both mutant and wild-type mtDNAs. During mitosis, the mtDNA is divided and stochastically distributed to the next generation. If the mutation load surpasses the threshold in the tissue, affected patients will present corresponding clinical manifestations (41). Since tissues with high demand for energy metabolism have lower thresholds, organs like brain, heart, skeletal muscle, optic nerve, and retina are more vulnerable to the pathogenic effects of mtDNA mutations (42). Other modifier factors including nuclear modifier genes (43), epigenetic phenomena (44), secondary mtDNA mutations (45), mitochondrial haplogroup (46), or environmental factors (47), also get involved in modifying the clinical expression and consequently cause differences in phenotypes among patients.

Similarly, additional factors may play a role in the phenotypic manifestation of the T12811C mutation as well. Here, all of our patients denied smoking and drinking alcohol, which were considered as the major environmental factors for LHON (47). However, in addition to T12811C, another variant G3946A was detected in case 1, although with relatively low heteroplasmy. The G3946A mutation can affect the assembly or turnover of complex I. It was reported to be associated with MELAS and hearing loss and the case 1 happened to have leukoencephalopathy and hearing loss as well (48). This mtDNA variant might interact with T12811C, triggering or exacerbating the expression of the phenotype. Meanwhile, all our patients harbored a single nucleotide polymorphism of A10398G, which was previously reported to increase the penetrance of primary G11778A mutation of LHON (49). However, due to its high frequency in Asian population (66%) (50), the effect of A10398G mutation on our patients remains unclear. Thus, there are still possibilities that the involved additional genetic and/or environmental factors were unrevealed in our patients.

## Conclusion

In summary, we reported four patients with T12811C mtDNA mutation presenting with various clinical phenotypes as LHON, hypertrophic cardiomyopathy, leukoencephalopathy, cochlea lesion, spinal cord lesion, peripheral neuropathies, and ophthalmoplegia. Mitochondrial disease should be considered as one of the differential diagnoses in patients manifesting with optic atrophy or ophthalmoplegia.

## Ethics Statement

The studies involving human participants were reviewed and approved by the Institutional Review Board of Eye, Ear, Nose, and Throat Hospital of Fudan University. Written informed consent was obtained from the individual(s), and minor(s)’ legal guardian/next of kin, for the publication of any potentially identifiable images or data included in this article.

## Author Contributions

YC and GT conceptualized and designed the study. GT recruited the patients and collected the data. CF and QC obtained patients’ consent, examined, and followed-up the patients. QX and PS analyzed the genetic testing results, reviewed the literature, and wrote the manuscript. XS analyzed the cases and revised the manuscript. All authors approved the final manuscript.

## Conflict of Interest

The authors declare that the research was conducted in the absence of any commercial or financial relationships that could be construed as a potential conflict of interest.

## Publisher’s Note

All claims expressed in this article are solely those of the authors and do not necessarily represent those of their affiliated organizations, or those of the publisher, the editors and the reviewers. Any product that may be evaluated in this article, or claim that may be made by its manufacturer, is not guaranteed or endorsed by the publisher.
